# Multiparametric MRI assessment of renal blood oxygenation, fat content, and hemodynamics in an animal model of metabolic dysfunction-associated steatotic liver disease

**DOI:** 10.3389/fendo.2025.1547016

**Published:** 2025-06-02

**Authors:** Chao Wang, Jiaming Qin, Hongtao Yuan, Jin Zhou, Ting Cao, Jinxia Zhu, Simeng Kang, Shuangshuang Xie, Wen Shen

**Affiliations:** ^1^ First Central Hospital of Tianjin Medical University, Tianjin, China; ^2^ Radiology Department, Hohhot First Hospital, Hohhot, Inner Mongolia, China; ^3^ Department of Radiology, Tianjin First Central Hospital, School of Medicine, Nankai University, Tianjin Institute of Imaging Medicine, Tianjin, China; ^4^ General Surgery Department, Affiliated Hospital of Inner Mongolia Minzu University, Tongliao, Inner Mongolia, China; ^5^ MR Research Collaboration, Siemens Healthineers, Beijing, China; ^6^ Department of Stomatology, Affiliated Hospital of Inner Mongolia Medical University, Hohhot, Inner Mongolia, China

**Keywords:** blood oxygenation, fat content, hemodynamics, MASLD, multiparametric MRI, renal

## Abstract

**Aims:**

Metabolic dysfunction-associated steatotic liver disease (MASLD) is a significant risk factor for chronic kidney disease. There is a lack of an accurate and comprehensive technique for detecting MASLD-related renal injury. This study aims to evaluate the efficacy of arterial spin labeling (ASL), blood oxygen level-dependent (BOLD) imaging, and proton density fat fraction (PDFF) for assessing renal injury in an animal model of MASLD.

**Methods:**

An animal model of MASLD was established using a high-fat diet. Forty-nine 6-week-old male Sprague-Dawley rats were divided into the pathology (14, 16, 18, 20, 22, and 24 weeks, n = 7 per subgroup) and continuous-scanning (n = 7) groups. Renal alterations at different time points were quantified through the application of ASL-renal blood flow (RBF), BOLD-T2*, and Fat Fraction (FF), alongside pathological indices and blood biochemical markers.

**Results:**

RBF did not change significantly from 14–24 weeks, consistent with the peritubular capillary density. Compared with those at week 14, renal T2* significantly decreased at week 20, FF increased at week 20, and serum creatine levels increased at week 24. Renal T2* and FF were significantly correlated with renal H&E scores and HIF-1α expression (|r| = 0.3552–0.7745). Kidney BOLD-T2*, liver and kidney FF enabled detecting renal injury in an animal model of MASLD (area under the curve = 0.76–0.86).

**Conclusion:**

During fatty liver disease progression, renal blood oxygen levels decreased, fat deposition increased, and blood flow remained unchanged. BOLD and PDFF allowed accurately quantifying these changes to facilitate early detection of kidney injury.

## 1 Introduction

Metabolic Dysfunction-Associated Steatotic Liver Disease (MASLD) has emerged as a major global health concern, driven by its rising prevalence and the severe consequences associated with its progression. MASLD exhibits a notably high prevalence, especially among middle-aged individuals who are overweight yet have normal liver enzyme levels (1). Furthermore, MASLD is linked to an elevated risk of cardiovascular diseases and metabolic disorders, as well as a growing risk of kidney disease (2, 3). MASLD has been identified as a significant risk factor for chronic kidney disease (CKD) (4). Abnormalities in cholesterol and fatty acid metabolism in individuals with fatty liver may contribute to the onset and progression of CKD.

Early detection of kidney injury is crucial for improving treatment outcomes, reducing complications, enhancing prognoses, and lowering healthcare burden (5). Kidney injury is primarily diagnosed based on serum creatinine (Scr) levels; however, Scr is not sensitive enough to detect early kidney injury (6). Novel blood and urine biomarkers lack standardized diagnostic criteria and sufficient clinical validation, limiting their application in clinical practice (7). Thus, new non-invasive diagnostic methods are urgently needed.

The potential pathophysiological mechanisms of fatty liver-associated renal injury include cellular hypoxia, lipid deposition, and inflammatory responses (8, 9). Previous studies have indicated that the primary factor contributing to renal injury is alterations in renal hemodynamics leading to changes in oxygenation status (10). However, whether this theory applies to kidney injury associated with fatty liver disease remains unclear. Additionally, recently developed lipid-targeted therapeutics are undergoing clinical trials for CKD (11), highlighting the critical importance of non-invasive methods for detecting renal lipid content. Blood oxygen level-dependent (BOLD) response is highly sensitive to tissue hypoxia and enables effectively assessing tissue oxygenation status (12). Arterial spin labeling (ASL) is a non-invasive and reliable technique for evaluating organ blood perfusion (13). Proton density fat fraction (PDFF) allows assessing renal lipid deposition and is correlated with histological fat quantification in renal tissue (14). Those techniques can be used to assess the progression of fatty liver-related renal injury. Therefore, we used ASL, BOLD, and PDFF imaging to assess the changes in renal blood flow (RBF), renal oxygenation, and lipid content in a rodent model of MASLD.

## 2 Methods

### 2.1 Intervention

**Figure 1:**
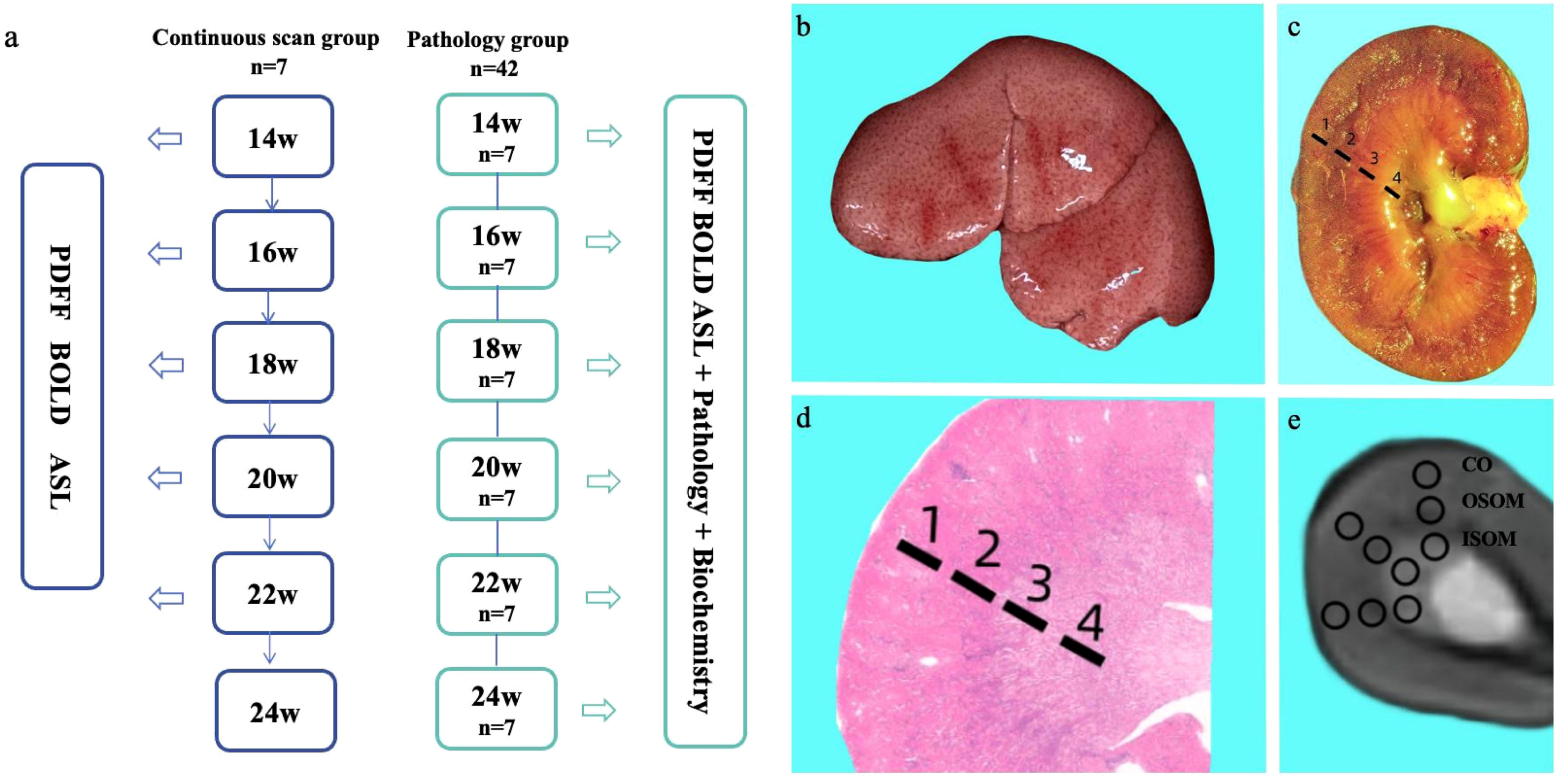
Experimental workflow **(a)**. Gross specimens of a rat liver after 14 weeks of consuming a high-fat diet **(b)**. Gross specimens of rat kidneys and schematic diagram of anatomical regions **(c)**: 1 = cortex; 2 = outer stripe of the outer medulla; 3 = inner stripe of the outer medulla; 4 = inner medulla. H&E-stained pathological sections of the kidney and corresponding schematic diagram of anatomical regions in rats on a high-fat diet **(d)**. Schematic diagram of ROI delineation in the T2-weighted axial fat suppression sequence of the kidney **(e)**.

Adult Sprague-Dawley rats weighing 160–200 g (Beijing HFK Bioscience Co. Ltd., Beijing, China) were used. All rats were housed in the Key Laboratory of Organ Transplantation, Tianjin, China. The rats had unrestricted access to food and water and were maintained on a 12-hour/12-hour light/dark cycle. All rats were fed a high-fat diet (D12451: 45 kcal% fat, 20 kcal% protein, 35 kcal% carbohydrate) (Xiao Shu You Tai Biotechnology Co., Ltd, Beijing, China). Forty-nine rats were divided into the continuous-scanning (n = 7) or pathology (n = 42) groups. In the continuous-scanning group, liver PDFF and kidney BOLD, PDFF, and ASL scans were performed at 14, 16, 18, 20, 22, and 24 weeks. The pathology group was further divided into six subgroups (n = 7 per subgroup) and fed a high-fat diet for 14, 16, 18, 20, 22, and 24 weeks. At each time point, the corresponding rats underwent liver PDFF and kidney BOLD, PDFF, and ASL examinations (**Figure 1a**). The body weights of all rats in both groups were monitored after the scans. In the pathology group, rats were euthanized after the scans at each time point, and venous blood was collected for biochemical analyses. Liver and kidney tissue samples were obtained for histopathological analyses.

### 2.2 Magnetic resonance imaging assessment

MRI scans were performed on a 3T MRI system (MAGNETOM Prisma, Siemens Healthineers, Erlangen, Germany) using a dedicated small animal coil (16 channels, Chenguang Medical Technology Co., Ltd., Shanghai, China). Rats were fasted for 8 hours prior to the MRI scan. During the scan, anesthesia was maintained using a small animal anesthesia machine (Yuyan Scientific Instrument Co., Ltd., Shanghai, China) with isoflurane (2%) at a flow rate of 0.6 L/min. The rats were scanned in the supine position to minimize respiratory artifacts. T2-weighted images were acquired using a turbo spin-echo sequence to observe the basic anatomical structure of the kidneys. BOLD images were acquired in the axial plane using a multi-echo gradient-echo sequence. The Dixon technique was used to obtain transverse liver and kidney images to calculate FF values. A research three-dimensional turbo-gradient spin-echo pulsed ASL sequence was performed to obtain RBF. Seven inversion times (300, 500, 700, 900, 1100, 1300, and 1500 ms) and a bolus length of 700 ms were used. **Table 1** presents the specific parameters of these sequences.

**Table 1:** MRI sequence parameters.

Sequences	TR (ms)	TE (ms)	FOV (mm^2^)	Slice thickness (mm)	Matrix	Acquisition time (min:s)
BOLD	255	3.22, 5.83, 8.42, 11.01, 13.63, 16.22	85×62	3	192×154	2:57
Dixon	3.93	1.3, 2.53	147×160	2	128×105	0:20
ASL	6000	49.86	153×153	3	64×64	7:05

TR, repetition time; TE, echo time; FOV, field of view.

### 2.3 Laboratory assessment

The rats were humanely euthanized at the respective time points, and 4 mL of venous blood was collected from the inferior vena cava. The blood samples were then centrifuged at 3500 rpm for 15 minutes at 4°C. The supernatant was collected and analyzed using the URIT-8210 automatic biochemical analyzer (URIT Medical Electronic Co. Ltd., Guangxi, Guilin, China).

All rats remained fasted and anesthetized after the MR scans. Blood glucose levels were measured using the Yasee GLM-77 glucometer with venous blood collected from the tail vein. In the pathology group, blood samples were collected from the inferior vena cava. The centrifuged serum samples were analyzed for alanine aminotransferase (ALT), aspartate aminotransferase (AST), triglycerides (TG), cholesterol (CHOL), high-density lipoprotein, Scr, and blood urea nitrogen (BUN) using corresponding reagent kits (Jisibiology, China).

All experiments were reviewed and approved by the animal care committee of the research institution. The study was approved by the ethics committee of Nankai University (2022-SYDWLL-000236).

### 2.4 Image analysis

Renal T2* and RBF maps were automatically generated inline after data acquisition. Hepatic and renal PDFF maps were calculated from Dixon images. Regions of interest (ROIs) were delineated on the MRI workstation (Siemens Healthineers, Erlangen, Germany) using post-processing tools (*syngo*.via, Siemens Healthcare, Erlangen, Germany) to compute T2*, FF, and RBF values. On liver PDFF images, ROIs were manually delineated, with each ROI area maintained at 5 mm^2^. T2* images derived from the BOLD sequence were referenced against T2-weighted images, and ROIs were delineated on the cortex (CO), outer stripe of the outer medulla (OSOM), and inner stripe of the outer medulla (ISOM) to obtain T2* values. Each ROI area was maintained at 3 mm^2^. On renal RBF images, ROIs were manually delineated to calculate corresponding values with each ROI area maintained at 3 mm^2^. ROIs were selected to avoid the renal artery and cover the largest cross-section of the renal anatomy. Each area was measured three times, and the average value was recorded. Two radiologists (with 5 and 10 years of experience in abdominal MRI) evaluated the MRI data following a double-blind protocol.

### 2.5 Histopathological and immunohistochemical assessments

After the MRI scans were completed, the rats in the pathology group were euthanized. A portion of the left liver lobe was fixed in 10% neutral buffered formalin (Guangzhou Vigus Biotechnology Co., Ltd., Guangzhou, China). The right kidney was excised and bisected longitudinally, then fixed in 10% neutral buffered formalin. After fixation, the liver and kidney tissues were routinely processed, embedded in paraffin, sectioned into approximately 2-mm-thick slices, and stained with hematoxylin and eosin (H&E Biossci BPO92). Liver tissues were also stained using Masson’s trichrome stain (Biossci BPO28). Kidney tissues underwent immunohistochemical staining for hypoxia-inducible factor-1α(HIF-1α, PTG 20960-1-AP) and CD34 (abcam, ab81289). H&E-stained sections were evaluated under a microscope, focusing on three fields of view (FOVs) in the CO, OSOM, and ISOM. The sections were scored based on the percentage of damage, with specific criteria, including tubular dilation, increased luminal mucus, tubular cell swelling, vacuolization, protein casts, interstitial vascular dilation and congestion, and tubular necrosis (0: normal; 1: ≤5%; 2: 5%–25%; 3: 25%–50%; 4: 50%–75%; 5: 75%–100%). The HIF-1α positive-expression percentage was assessed, and the CD34 positive-expression percentage was used to evaluate peritubular capillary (PTC) density. These assessments were performed using ImageJ open-source software for semi-quantitative analysis. Five random areas were selected from each section for statistical analysis, with three FOVs per area, and the average values were calculated. Two readers, with 8 and 5 years of clinical pathology experience, evaluated the sections independently, blinded to the experimental conditions.

### 2.6 Statistical analysis

GraphPad Prism (version 9.5.1; GraphPad Software, LLC) and HIPLOT (version Pro 2.0; https://hiplot.com.cn/) were used for statistical analysis. All measured data are presented as means ± standard deviation. Differences in RBF, T2* and FF among time points in the continuous-scanning group were assessed using one-way repeated-measures (ANOVA) for normally distributed data or the Kruskal-Wallis test for non-normally distributed data. Histological differences among time points in the pathology group were evaluated using one-way ANOVA for normally distributed data or the Kruskal-Wallis test for non-normally distributed data. Correlations between MRI parameters, histopathological indexes and biochemical markers were assessed using Pearson’s correlation for normally distributed data or Spearman’s correlation for non-normally distributed data. Using an H&E score of ≥1 as the threshold, samples were categorized as injured kidneys or normal kidneys. The diagnostic performances of kidney T2*, liver and kidney FF, and Scr for renal injury were evaluated using receiver operating characteristic (ROC) curve analysis. P < 0.05 was considered statistically significant.

## 3 Results

### 3.1 General observation of the model

**Figure 2:**
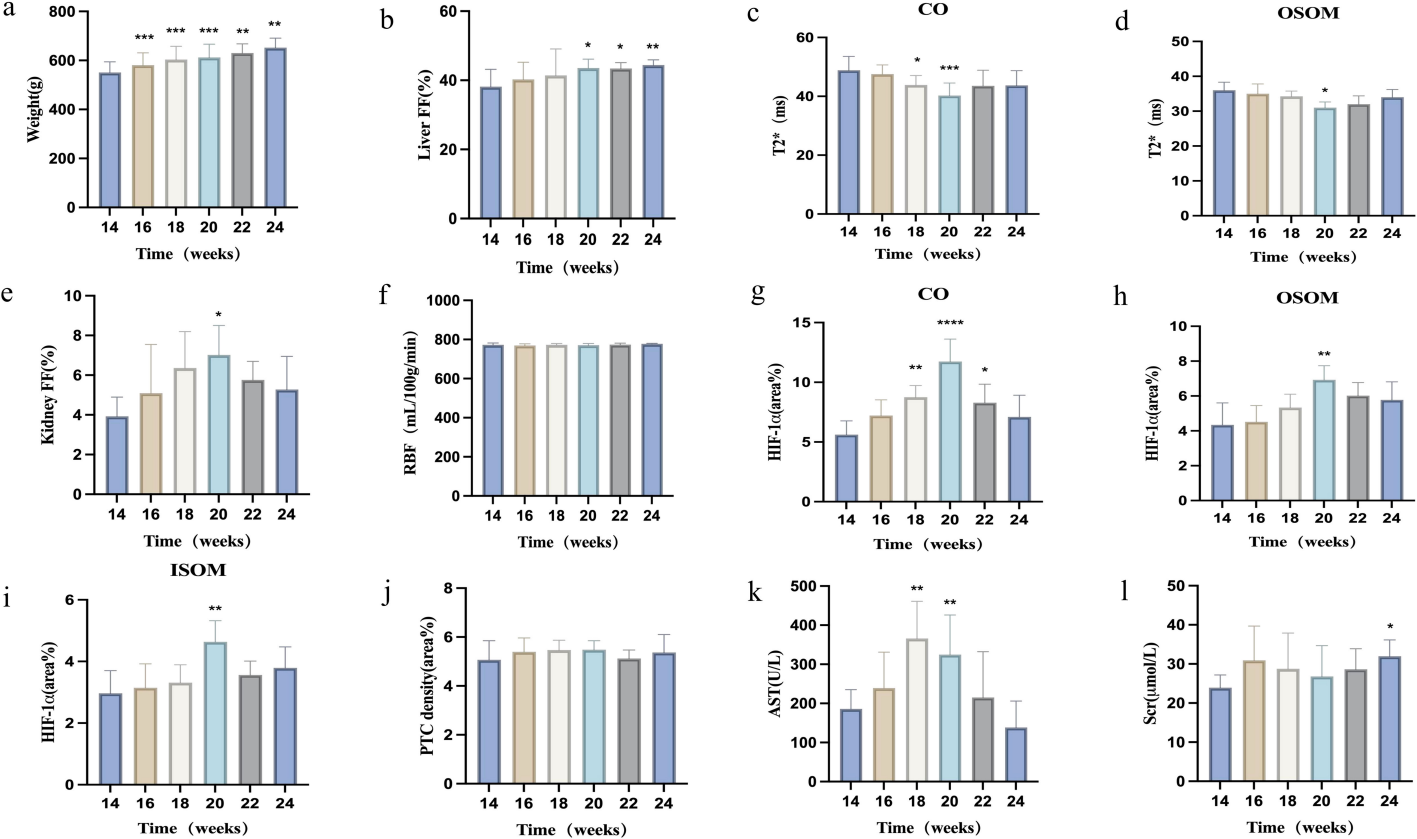
Changes in various parameters in rats after 14 weeks of consuming a high-fat diet. **(a)** Body weight; **(b)** Liver FF changes over time; **(c, d)** BOLD-T2* changes in renal cortex and outer stripe of the outer medulla over time; **(e, f)** Renal FF and RBF changes over time; **(g–j)** Immunohistochemical staining indices (HIF-1α, PTC density) in the renal cortex, outer stripe of the outer medulla, and inner stripe of the outer medulla over time; **(k, l)** Biochemical indices (AST, Scr) changes over time. *P < 0.05; **P < 0.01, compared with baseline (14 weeks).

**Figure 1b** shows a gross fatty liver specimen, characterized by diffuse enlargement, a gray-yellow and rough surface, thick and blunt edges, and depressions upon pressure. **Figure 1c** shows the gross specimen of a kidney from a rat with fatty liver. **Figure 1d** shows H&E-stained kidney sections with the CO, OSOM and ISOM. **Figure 1e** is a T2-weighted fat suppression image with its corresponding ROI diagram, with anatomical bands corresponding to the pathological sections of the kidney. Body weights did not significantly differ between the continuous-scanning and pathological groups (P = 0.4010). The body weights of the rats significantly increased from 14 to 24 weeks (**Figure 2a**).

### 3.2 MRI measurements

#### 3.2.1 Liver and renal FF

The hepatic FF increased progressively, whereas the renal FF initially increased, then decreased. Liver and renal FF values did not significantly differ between the two groups (all P > 0.05). The hepatic FF increased significantly starting at 20 weeks compared with that at 14 weeks, then peaked at 24 weeks (all P < 0.05). Renal FF exhibited the most significant increase at 20 weeks (P < 0.05), followed by a gradual decrease (**Figures 2b, e**, **Table 2**).

**Table 2:** MRI parameters for the continuous-scanning and pathological groups at different time points.

Parameters	Groups	Renal zones	14 Weeks	16 Weeks	18 Weeks	20 Weeks	22 Weeks	24 Weeks
Liver FF (%)	CG		38.19 ± 5.01	40.29 ± 4.96	41.43 ± 7.66	43.57 ± 2.57*	43.43 ± 1.72*	44.43 ± 1.51**
	PG		41.34 ± 2.24	39.00 ± 4.00	43.86 ± 6.57	48.71 ± 3.86**	45.57 ± 6.92	44.71 ± 2.75
Kidney T2* (msec)	CG	CO	48.86 ± 4.74	47.57 ± 3.10	43.86 ± 3.24*	40.29 ± 4.23***	43.57 ± 5.32	43.71 ± 5.02
		OSOM	36.00 ± 2.31	35.00 ± 2.83	34.29 ± 1.50	31.00 ± 1.63*	32.00 ± 2.38	34.00 ± 2.24
		ISOM	43.29 ± 5.59	41.43 ± 4.83	39.43 ± 3.31	37.57 ± 4.28	39.29 ± 3.99	38.86 ± 3.24
	PG	CO	50.09 ± 4.18	51.16 ± 2.99	44.82 ± 4.42**	40.88 ± 2.41***	48.86 ± 1.57	43.86 ± 4.10**
		OSOM	35.82 ± 2.19	35.72 ± 1.61	34.16 ± 2.08	31.33 ± 3.05**	36.57 ± 1.81	34.29 ± 2.69
		ISOM	41.54 ± 3.57	37.29 ± 4.61	35.71 ± 4.89*	33.71 ± 3.82*	44.00 ± 2.58	41.29 ± 5.23
Kidney FF (%)	CG		3.93 ± 0.97	5.09 ± 2.45	6.37 ± 1.83	7.02 ± 1.48*	5.76 ± 0.94	5.28 ± 1.66
	PG		4.08 ± 1.27	3.47 ± 1.14	5.76 ± 1.22*	6.45 ± 1.39**	5.34 ± 1.00	5.90 ± 1.84
Kidney RBF (mL/100g/minute)	CG		773.14 ± 9.16	769.71 ± 8.14	773.29 ± 6.40	770.86 ± 8.73	773.86 ± 7.31	777.57 ± 2.88
	PG		775.29 ± 6.32	775.00 ± 7.05	775.86 ± 4.41	776.00 ± 5.26	776.86 ± 4.34	773.86 ± 8.15

FF, fat fraction; RBF, renal blood flow; CG, continuous-scanning group; PG, pathological group; CO, cortex; OSOM, outer stripe of the outer medulla; ISOM, inner stripe of the outer medulla. *P < 0.05; **P < 0.01; ***P < 0.001 vs. baseline (14 w).

#### 3.2.2 Renal BOLD-T2*

Renal T2* values initially decreased, then recovered slightly. T2* values did not significantly differ among renal anatomical zones between the two groups (all P > 0.05). Compared with baseline, renal cortical T2* showed the most significant decreases at 18 and 20 weeks, reaching its lowest point at 20 weeks (P < 0.05). The T2* value in the renal OSOM dropped to its lowest value at 20 weeks, then gradually recovered (P < 0.05; **Figures 2c, d**, **Table 2**).

#### 3.2.3 Renal ASL

**Figure 3:**
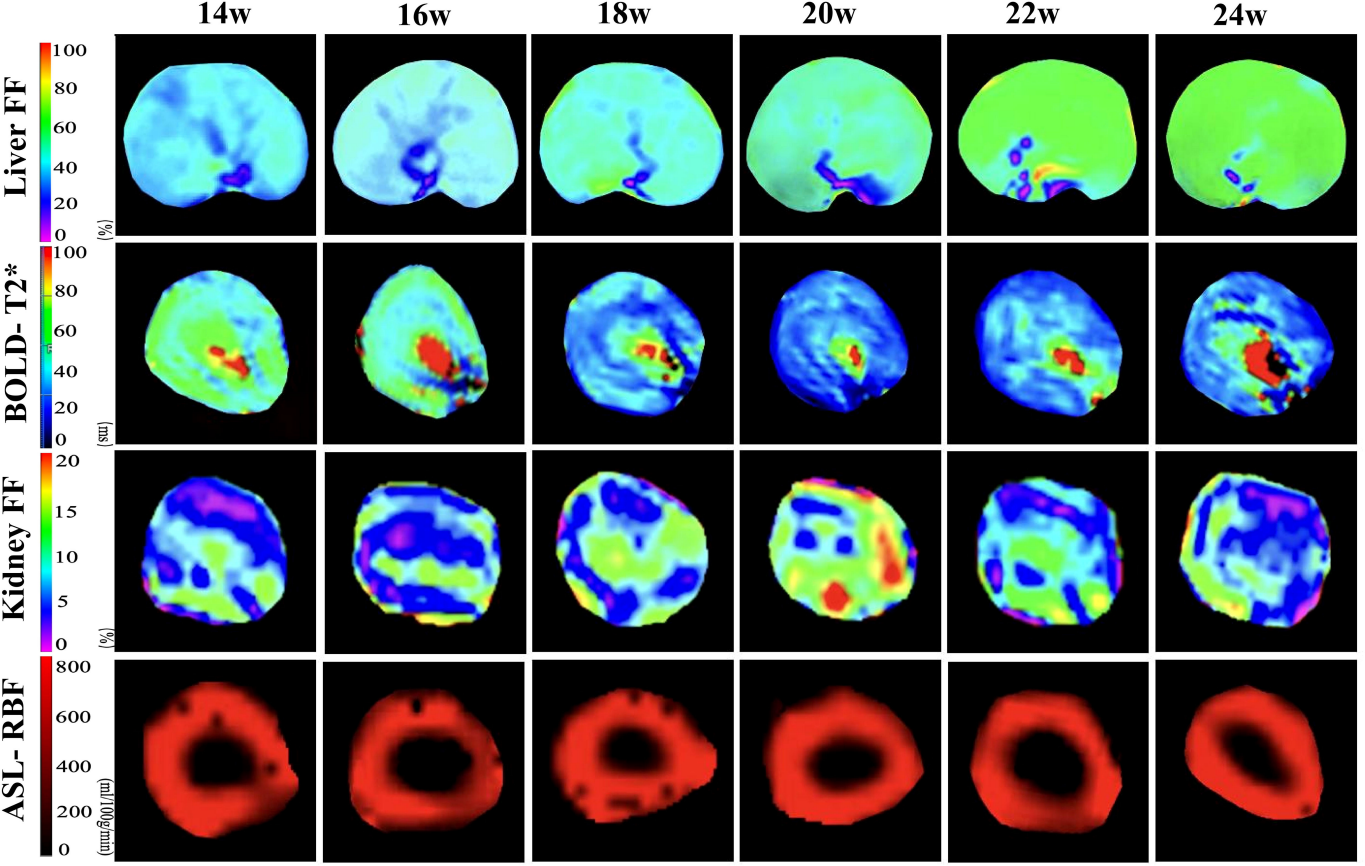
Pseudo-color images of hepatic FF and renal BOLD-T2*, FF, and ASL-RBF changes from 14–24 weeks of consuming a high-fat diet. The liver FF images demonstrate a gradual increase in FF values from 14–24 weeks. The BOLD images indicate a decline in T2* values starting at 18 weeks, with the most significant decrease at 20 weeks, followed by a slight recovery from 20–24 weeks. The kidney FF images show the most pronounced increase in FF values at 20 weeks, followed by a decrease from 20–24 weeks. The ASL images reveal no significant changes in RBF values from 14–24 weeks.

Renal cortical ASL did not significantly differ between the two groups (all P > 0.05). The renal cortical RBF did not significantly differ at any time points (**Table 2**, **Figure 2f**). **Figure 3** illustrates the trend in pseudo-color changes for various MRI parameters.

### 3.3 Histopathological indices

Liver H&E staining showed numerous lipid vacuoles in the hepatocytic cytoplasm, with nuclei pushed to one side and varying degrees of inflammatory cell infiltration in the portal area. The number of large vacuoles increased over time. Masson staining of the liver showed no significant fibrosis around hepatocytes or in the portal area throughout the study period.

H&E-stained kidney sections showed no significant damage at 14 weeks (baseline group). From 16–20 weeks, renal tubular cells gradually became vacuolated, the tubular lumen slowly expanded, and mucus increased within the lumen, with the most significant damage at 20 weeks. After 20 weeks, damaged renal tubular cells began to partially recover.

HIF-1α was positively expressed in all renal anatomical regions at different time points, exhibiting an overall trend of an initial increase followed by a decrease. Compared with that at baseline, HIF-1α expression in the CO and OSOM was most significantly elevated at 20 weeks (all P < 0.05), and HIF-1α expression in the ISOM was most significantly elevated at 18 and 20 weeks (P < 0.05).

**Figure 4:**
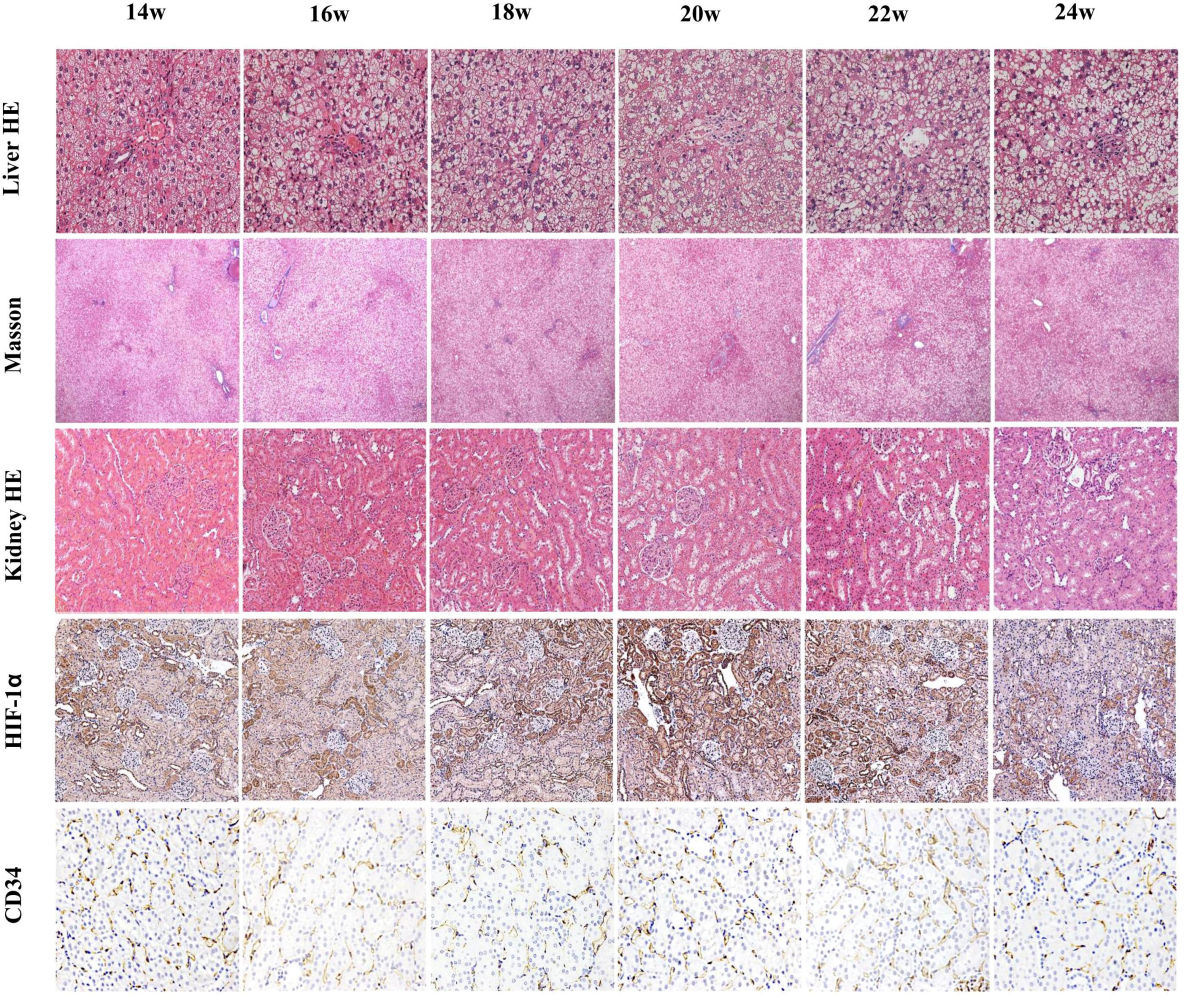
Liver H&E staining (×200), liver Masson trichrome staining (×40), kidney H&E staining (×200), HIF-1α staining (×200), and CD34 staining (×200). Liver H&E staining shows an increasing degree of hepatocellular steatosis and an increase in large fat droplets after 14 weeks of consuming a high-fat diet. Masson staining shows no significant fibrosis around hepatocytes throughout the process. Renal H&E staining shows no obvious damage at 14 weeks, slight vacuolation of tubular cells at 16 weeks, and gradually pronounced vacuolation at 18 weeks, with the most severe damage at 20 weeks, and partial recovery after tubular cell damage from 22–24 weeks. Renal HIF-1α staining indicates varying degrees of hypoxia at different timepoints, with the most severe hypoxia at 20 weeks, which gradually decreased over time. CD34 staining shows no significant change in the peritubular capillary density of the kidneys.

PTC density did not significantly differ at any time points (P > 0.05). **Figure 4** illustrates the pathological indicators. **Figures 2g–j** and **Table 3** display the values and trends in parameters.

**Table 3:** Pathological and immunohistochemical scores at different time points.

Pathological Indicators	Renal zones	14 Weeks	16 Weeks	18 Weeks	20 Weeks	22 Weeks	24 Weeks
H&E score	CO	0 ± 0	0.29 ± 0.49	0.57 ± 0.54	2.29 ± 0.49***	1.86 ± 0.38***	1.71 ± 0.49**
	OSOM	0 ± 0	0.29 ± 0.49	0.57 ± 0.54	1.86 ± 0.38***	1.29 ± 0.49**	1.71 ± 0.49***
	ISOM	0 ± 0	0 ± 0	0.14 ± 0.38	1.14 ± 0.38***	1.00 ± 0.58**	1.14 ± 0.38***
HIF-1α	CO	5.62 ± 1.15	7.22 ± 1.31	8.76 ± 0.97**	11.75 ± 1.88****	8.30 ± 1.55*	7.11 ± 1.81
	OSOM	4.35 ± 1.26	4.52 ± 0.94	5.33 ± 0.77	6.92 ± 0.81**	6.02 ± 0.75	5.78 ± 1.03
	ISOM	2.97 ± 0.74	3.15 ± 0.77	3.31 ± 0.58	4.64 ± 0.69**	3.56 ± 0.45	3.79 ± 0.68*
PTC		5.07 ± 0.78	5.39 ± 0.57	5.47 ± 0.40	5.48 ± 0.37	5.12 ± 0.35	5.37 ± 0.73

H&E, hematoxylin-eosin; HIF-1α, hypoxia-inducible factor-1α; PTC, peritubular capillary; CO, cortex; OSOM, outer stripe of the outer medulla; ISOM, inner stripe of the outer medulla.*P < 0.05; **P < 0.01; ***P < 0.001 vs. baseline (14 w).

### 3.4 Laboratory tests

Fasting blood glucose levels exhibited an overall increasing trend. Compared with those at baseline (14 weeks), fasting blood glucose levels increased significantly at 18 and 20 weeks (P < 0.05), with the most significant increase at 24 weeks (P < 0.001). ALT, AST, and CHOL levels were initially elevated, then subsequently declined. Compared with those at baseline, ALT and AST levels increased significantly at 18 and 20 weeks (all P < 0.05). CHOL levels increased significantly at 18 weeks (P < 0.05). TG levels initially increased, then decreased, then increased again, then finally decreased. TG levels increased significantly at 16 and 22 weeks (P < 0.05). Scr levels showed no significant changes from 14–22 weeks, but significantly decreased at 24 weeks compared with baseline (P < 0.05). BUN levels showed no significant changes from 14–24 weeks (P > 0.05). **Figures 2k, i** display the trends in AST and Scr, respectively. **Supplementary Table 1** provides the specific values for each parameter.

### 3.5 Correlations among MRI parameters, histopathology, and biochemical indicators

**Figure 5:**
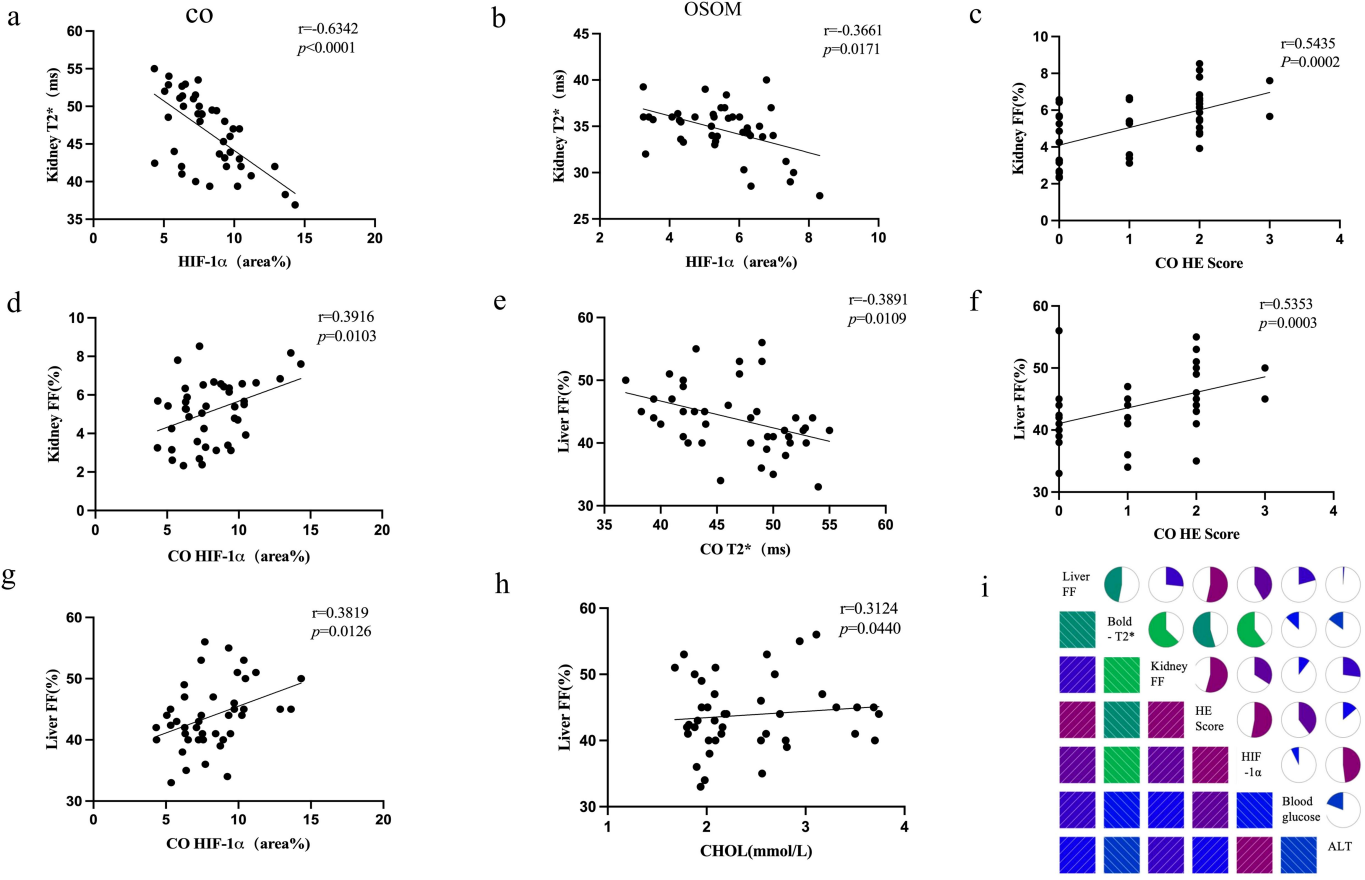
Correlation plots **(a–h)** depict the relationships among key biomarkers. The correlation matrix plot **(i)** illustrates the correlations between MRI parameters (T2*, FF), histopathological markers (H&E, HIF-1α), and one biochemical marker (ALT).

Renal T2* values in the CO and OSOM were negatively correlated with renal H&E scores and HIF-1α expression (CO: r = −0.6342 to −0.5461; OSOM: r = −0.3846 to −0.3552). T2* values in the ISOM were negatively correlated with ALT (r = −0.3255). Renal PDFF was positively correlated with H&E scores and HIF-1α expression across all anatomical regions (CO: r = 0.3916–0.5435; OSOM: r =0.5455–0.7745; ISOM: r =0.3925–0.4623). Renal T2* values in the CO were negatively correlated with liver PDFF (r = −0.3891). Blood glucose levels were positively correlated with renal H&E scores across all anatomical regions (r =0.3746–0.4111). Liver PDFF was positively correlated with CHOL (r = 0.3124). **Figures 5a–i** and **Supplementary Table 2** present the results and trends in these correlations.

### 3.6 ROC curve

**Figure 6:**
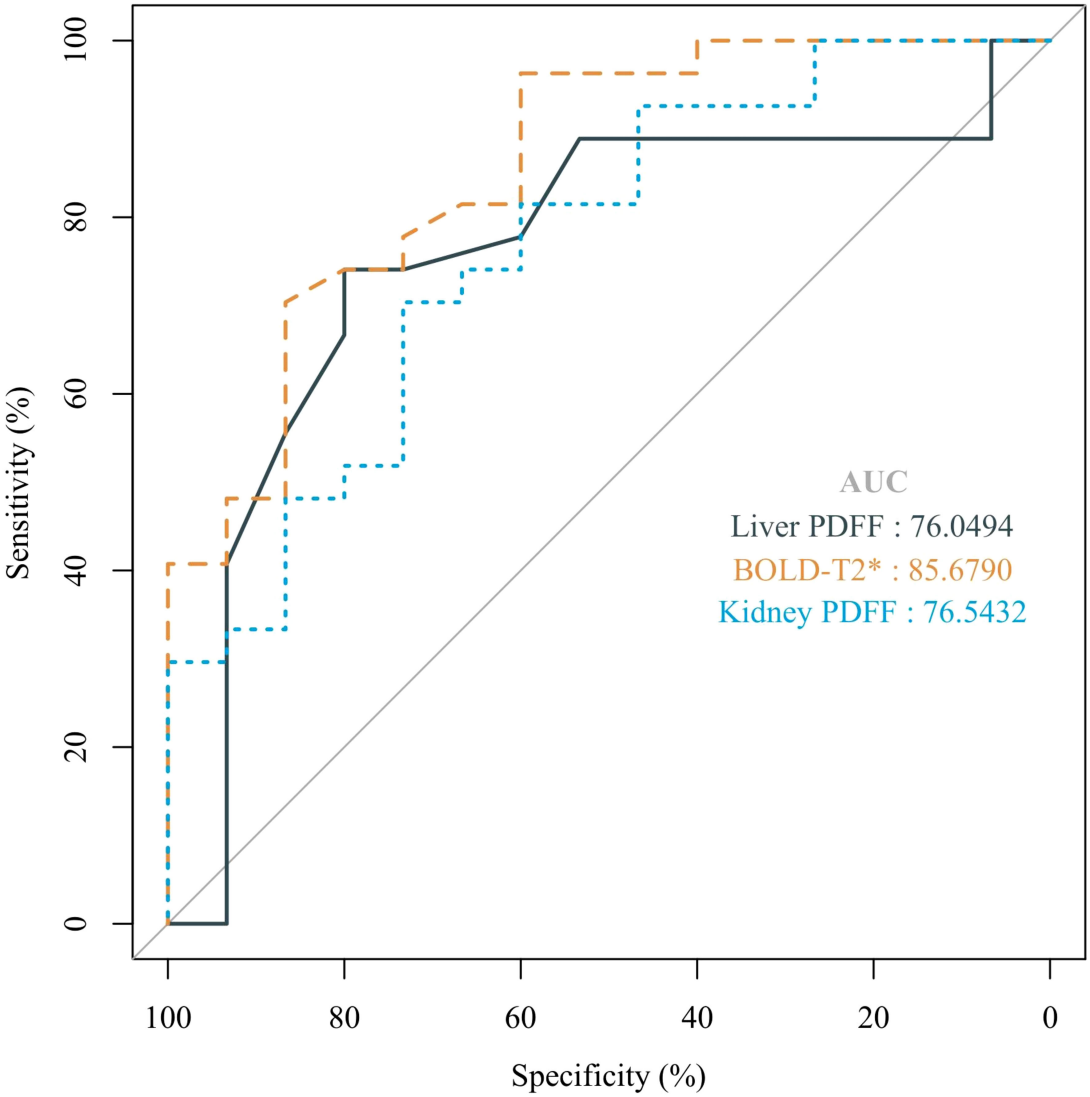
The ROC curves for liver FF, BOLD-T2*, and kidney FF were used to distinguish renal injury from non-renal injury in a rat model of MASLD from 14 weeks to 24 weeks.

ROC curve analysis demonstrated that renal BOLD-T2*, renal FF and liver FF detected renal injury with AUCs of 0.857, 0.764 and 0.761, respectively, whereas Scr could not be used to diagnose renal injury in this model (P > 0.05). Renal cortical T2* values showed the highest diagnostic efficacy, with 70.4% sensitivity and 86.7% specificity when <47.5 ms was used as the cutoff value (**Figure 6**, **Supplementary Table 3**).

## 4 Discussion

We found that from weeks 14 to 24 of feeding rats a high-fat diet, RBF remained unchanged, blood oxygen levels decreased, and the fat content increased. The alterations in BOLD-T2* and FF corresponded with histopathological changes and can be used to detect renal injury.

Liver H&E staining revealed that hepatic steatosis gradually increased over time throughout the modeling process, indicating a progressive rise in liver fat content. The liver FF also progressively increased, with the most pronounced increase occurring between 16 and 22 weeks, reflecting the continuous accumulation of TGs within the hepatic tissue. Masson staining of the liver showed no significant fibrosis around hepatocytes or in the portal areas. These findings confirm that the fatty liver model was successfully established and remained at the steatosis stage throughout the experiment, even at week 24, without progressing to fibrosis. We found that despite significant hepatic steatosis at week 14 as indicated on the H&E-stained liver sections, the renal H&E staining showed no noticeable damage. Therefore, we designated week 14 of the high-fat diet as the baseline timepoint for studying changes in the kidneys of rats with fatty liver.

Scr and BUN levels showed no significant increase during the modeling process, which is consistent with previous research findings (15). This might be because abnormalities in Scr levels typically become evident only when over half of the nephron units are damaged (16). At week 20, kidney FF and T2* values differed from those at baseline. Therefore, FF and T2* were more sensitive indicators of damage than were Scr and BUN. ASL measurements did not significantly differ between groups, suggesting that RBF did not change significantly throughout the study period. This was further supported by the lack of differences in PTC among the groups. The decreased T2* and increased HIF-1α indicate renal hypoxia. Because the RBF did not change significantly, this hypoxia was more likely due to increased oxygen consumption, consistent with findings in a rabbit model of diabetic kidney disease (DKD) (17), rather than a reduced oxygen supply. The increased compensatory oxygen demand observed despite stable RBF may be attributed to lipotoxicity-induced mitochondrial damage, which disrupts cellular energy metabolism. This damage disrupts the electron transport chain (ETC), leading to an overproduction of mitochondrial reactive oxygen species (mtROS) and a reduction in ATP synthesis (18, 19). Consequently, the increased energy demand further elevates the need for oxygen within the kidney. Renal H&E staining showed the most significant injury at 20 weeks, when HIF-1α staining was the most prominently positive. Subsequently, varying degrees of recovery occurred between 20 and 24 weeks, which may suggest that MASLD-related renal injury is a reversible process. H&E staining of the kidneys revealed tubular vacuolation and positive HIF-1α staining primarily in the CO and OSOM. These areas of damage correspond to the predominant distribution sites of glomeruli and proximal tubules. This may be related to the more pronounced impact of lipid deposition on the glomeruli and proximal tubules than on other renal compartments (11).

The inverse relationship between liver FF and renal T2* suggests systemic metabolic dysregulation, which may link hepatic steatosis to renal injury. Hepatic steatosis can trigger systemic inflammation, contributing to liver and kidney dysfunction in conditions like MASLD (20). Additionally, imbalances in lipid metabolism and oxidative stress exacerbate damage in both organs, with disrupted regulatory pathways promoting steatosis in the liver and oxidative stress in the kidneys (21–23). Renal T2*, H&E staining scores, and HIF-1α levels were strongly correlated which is characterized by increased oxidative stress and mitochondrial dysfunction leading to hypoxia. Yamamoto et al. (19) found that a high-fat diet led to accumulation of phospholipids within enlarged lysosomes in proximal tubule cells, accompanied by impaired autophagic flux. The relationship between mitochondrial oxidative stress, reactive oxygen species generation, and mitochondrial engulfment is intricately intertwined and involves various pathological conditions of acute kidney injury (18).

FF enabled quantitatively measuring the fat content in the renal parenchyma and was positively correlated with H&E scores and HIF-1α. We hypothesize that excessive lipid accumulation within renal cells may induce endoplasmic reticular stress and reactive oxygen species production, leading to mitochondrial dysfunction and impairing the cell’s ability to use oxygen. Liver FF values were positively correlated with serum CHOL levels, whereas renal FF was poorly correlated with lipid markers. This is consistent with previous research indicating that hyperlipidemia and intrinsic renal lipid metabolic regulatory mechanisms influenced renal ectopic lipid accumulation (14, 24, 25). Liver and renal FF were not correlated with serum TG levels, which is consistent with previous findings (26, 27). This lack of correlation may be because PDFF specifically reflects the TG concentration within the tissue rather than in the bloodstream. Consequently, while PDFF is a valuable tool for assessing tissue fat content, it may not directly represent systemic lipid metabolism or circulating TG levels.

ROC analysis revealed that renal BOLD-T2 and liver and kidney FF enable effectively diagnosing renal injury in a rat model of MASLD, whereas Scr is not a reliable diagnostic marker for renal injury in this model. These findings highlight the limitations of traditional biochemical markers such as Scr, which often fail to detect early renal changes associated with hypoxia and lipid deposition. By precisely quantifying these alterations, MRI techniques enable earlier and more reliable detection of renal injury, thus potentially improving clinical outcomes for patients with MASLD-related renal damage. Notably, Wang et al. also highlighted the diagnostic efficiency of cortical R2* and FF values, showing significant differences between healthy controls and CKD patients, thus supporting the potential of these imaging modalities for noninvasive renal function evaluation in clinical settings (28).

This study had several limitations. First, the lack of differentiation between the cortex and medulla in FF measurements may limit the ability to detect localized lipid accumulation patterns. Higher-field MRI, such as 7T, provides better signal-to-noise ratio, spatial resolution, and spectral accuracy, allowing for improved anatomical differentiation, more accurate fat quantification, and reduced partial volume effects in heterogeneous regions. Second, owing to the relatively low renal fat content, current standard histopathological staining methods cannot clearly show renal FF. Finally, high-fat diet models are effective at inducing hepatic steatosis but often fail to replicate more advanced features of human MASLD, such as fibrosis, which limits their relevance for translational research. As a result, these models may overestimate the effectiveness of drugs aimed at early-stage steatosis while underestimating the potential of therapies targeting fibrosis regression.

In conclusion, MASLD-related kidney injury in rats was associated with renal hypoxia and lipid deposition, but with no RBF decrease. BOLD and PDFF can serve as noninvasive methods of evaluating renal changes and detecting early renal injury. Integrating BOLD and PDFF into clinical practice can significantly enhance the monitoring and management of MASLD-related renal injury.

## Data Availability

The original contributions presented in the study are included in the article/**Supplementary Material**. Further inquiries can be directed to the corresponding authors.
