# Supplementary material for: Multiparametric MRI assessment of renal blood oxygenation, fat content, and hemodynamics in an animal model of metabolic dysfunction-associated steatotic liver disease
Source: Front Endocrinol (Lausanne). 2025 Jun 2;16:1547016. doi: 10.3389/fendo.2025.1547016 (PMC12171186; doi:10.3389/fendo.2025.1547016)
Supplement: Supplementary file 1 [file Table1.docx]

**Supplementary Table 1.** Serum Biochemical Indicators at Different Time Points

|  | | 14 Weeks | 16 Weeks | 18 Weeks | 20 Weeks | 22 Weeks | 24 Weeks |
| --- | --- | --- | --- | --- | --- | --- | --- |
| Blood glucose | CG | 7.10±0.46 | 7.54±0.59 | 8.80±1.17* | 8.91±1.49* | 8.24±1.40 | 10.21±0.79*** |
| (mmol/L) | PG | 6.96±0.88 | 7.36±0.77 | 7.94±0.96 | 7.13±0.70 | 9.50±1.58*** | 10.06±0.71*** |
| ALT (U/L) | | 98.81±41.47 | 156.24±114.71 | 327.76±131.24** | 213.83±68.29* | 182.89±141.90 | 108.94±78.04 |
| AST (U/L) | | 186.16±49.14 | 239.56±91.41 | 365.94±94.97** | 324.93±101.06** | 215.01±117.15 | 138.39±67.85 |
| ALT/AST | | 0.51±0.11 | 0.61±0.18 | 0.92±0.37** | 0.68±0.14 | 0.78±0.17* | 0.73±0.27 |
| TG (mmol/L) | | 0.91±0.19 | 1.32±0.46* | 1.08±0.31 | 1.20±0.37 | 1.32±0.20* | 0.99±0.49 |
| CHOL (mmol/L) | | 2.16±0.38 | 2.35±0.58 | 3.11±0.62* | 2.69±0.73 | 2.18±0.41 | 2.08±0.24 |
| HDL (mmol/L) | | 0.83±0.16 | 0.94±0.12 | 0.93±0.28 | 1.01±0.23 | 0.80±0.13 | 0.91±0.15 |
| TG/HDL | | 1.12±0.21 | 1.46±0.63 | 1.31±0.68 | 1.29±0.68 | 1.68±0.22 | 1.19±0.78 |
| Scr (μmol/L) | | 23.95±3.23 | 30.94±8.73 | 28.78±9.12 | 26.83±7.84 | 28.63±5.28 | 31.98±4.17* |
| BUN (mmol/L) | | 5.42±0.59 | 5.57±1.27 | 4.83±1.02 | 4.91±0.52 | 4.92±0.61 | 5.45±0.86 |

CG: continuous-scanning group; PG: pathological group; ALT: alanine aminotransferase; AST: aspartate aminotransferase; Scr: serum creatinine, BUN: blood urea nitrogen; TG: triglyceride; CHOL: cholesterol; HDL: high density lipoprotein. **P* < 0.05; ***P* < 0.01; ****P* < 0.001 vs. baseline (14 w)

‘

**Supplementary Table 2.** Correlations Between RBF and T2* and Relevant Indicators of Pathology and Immunohistochemistry

|  |  | H&E Score | | HIF-1α | | Liver FF | |
| --- | --- | --- | --- | --- | --- | --- | --- |
|  |  | r | P | r | P | r | P |
| Liver FF | CO | 0.5353 | 0.0003 | 0.3819 | 0.0126 |  |  |
|  | OSOM | 0.5431 | 0.0002 | 0.5592 | 0.0001 |  |  |
|  | ISOM | 0.5141 | 0.0005 | 0.4513 | 0.0027 |  |  |
| Kidney T2* | CO | −0.5461 | 0.0002 | −0.6342 | <0.0001 | −0.3891 | 0.0109 |
|  | OSOM | −0.3552 | 0.0210 | −0.3846 | 0.0119 | −0.2082 | 0.1858 |
|  | ISOM | 0.0143 | 0.9282 | −0.1743 | 0.2696 | −0.0674 | 0.9128 |
| Kidney FF | CO | 0.5435 | 0.0002 | 0.3916 | 0.0103 | 0.2391 | 0.1272 |
|  | OSOM | 0.5455 | 0.0002 | 0.7745 | <0.0001 |  |  |
|  | ISOM | 0.4623 | 0.0021 | 0.3925 | 0.0101 |  |  |
| Blood glucose | CO | 0.3746 | 0.0145 | −0.0910 | 0.5666 | 0.1510 | 0.3400 |
|  | OSOM | 0.3656 | 0.0173 | 0.0807 | 0.6114 |  |  |
|  | ISOM | 0.4111 | 0.0068 | 0.0430 | 0.7869 |  |  |

PDFF: proton density fat fraction; H&E: hematoxylin-eosin; HIF-1α: hypoxia-inducible factor-1α; PTC: peritubular capillary; Scr: serum creatinine; CO: cortex; OSOM: outer stripe of the outer medulla; ISOM: inner stripe of the outer medulla.

**Supplementary Table 3.** AUC, Sensitivity, Specificity, and Best Cut-Off Value for Liver FF, BOLD-T2*, Kidney FF

|  | Liver FF | BOLD-T2* | Kidney FF |
| --- | --- | --- | --- |
| Sensitivity (%) | 74.47 | 70.37 | 70.37 |
| Specificity (%) | 80.00 | 86.67 | 73.33 |
| AUC | 0.760 | 0.857 | 0.765 |
| Best Cut-off Value | >42.7 (%) | <47.5 (ms) | >5.27 (%) |

AUC: area under the curve
